# Menin Inhibition in Acute Myeloid Leukemia: Pathobiology, Progress and Promise

**DOI:** 10.3390/biomedicines14010219

**Published:** 2026-01-20

**Authors:** Utsav Joshi, Rory M. Shallis

**Affiliations:** Department of Malignant Hematology, Moffitt Cancer Center, Tampa, FL 33612, USA; utsav.joshi@moffitt.org

**Keywords:** acute myeloid leukemia, AML, *KMT2A*, menin, *NPM1*, *NUP98*

## Abstract

Acute myeloid leukemia (AML) is a highly aggressive malignancy defined by significant biological diversity and variable patient outcomes. A key subset of AML is driven by abnormalities that lead to the overexpression of the oncogenic transcription factors HOXA9 and MEIS1. These abnormalities include *KMT2A* (formerly *MLL*) rearrangements and *NPM1* mutations, as well as other rare lesions such as *NUP98* rearrangements. This review focuses on the biology of the *KMT2A*, *NPM1*, and *HOX/MEIS1* pathways, dissecting their molecular mechanisms of leukemogenesis. A central theme is the role of the scaffolding protein menin in the epigenetic regulation of this pathway, which ultimately drives malignant transformation. Currently, the clinical landscape is being transformed by the emergence of menin inhibitors as promising therapeutic agents for AML harboring these specific genetic anomalies. We evaluate the latest data on various menin inhibitors—both as monotherapy and in combinations—emphasizing their efficacy and safety profiles. As new evidence continues to accumulate with recent drug approvals and ongoing randomized, phase 3 studies, menin inhibitors are rapidly becoming a component of the AML treatment paradigm for relapsed/refractory and likely newly diagnosed disease.

## 1. Introduction

Acute myeloid leukemia (AML) is a clonal disorder of hematopoietic progenitors characterized by impaired maturation, expansion of leukemic blasts, and suppression of normal hematopoiesis [1]. Historically, treatment of AML relied largely on the use of chemotherapy for all patients irrespective of a disease biology that, amongst human tumors that have been extensively appraised, is one of the most heterogeneous. Advances in molecular genetics and disease mechanisms have reshaped this model, opening multiple avenues for targeted therapies. Among these, menin inhibitors have emerged as a promising option for AML driven by histone-lysine N-methyltransferase 2A (*KMT2A*) rearrangements (*KMT2A-r*), nucleophosmin 1 *(NPM1*) mutations, and other rarer lesions characterized by oncogenic homeobox (*HOX*) *A9*/myeloid ecotropic virus insertion site 1 gene (*MEIS1*) overexpression (e.g., *NUP98* rearrangements, *UBTF* tandem duplications, *RUNX1/SETD1* or *CEPBA/RUNX1* co-mutations) [2]. This review summarizes the pharmacologic basis of menin inhibition, examines current therapeutic strategies, and offers a perspective on future directions for treating this distinct form of AML.

## 2. KMT2A Function and Aberrancy

*KMT2A*, formerly known as mixed-lineage leukemia (*MLL*), is central to normal hematopoiesis, and its rearrangements account for roughly 3–6% of adult *de novo* AML and up to 10% of cases arising out of prior cytotoxic therapy [3]. The *KMT2* family includes a group of methyltransferases that catalyzes the methylation of histone three lysine 4 (H3K4) through their conserved *SET* domain (Su(var)3–9, Enhancer of zester and Trithorax), playing an important role in epigenetic regulation [4]. The mature *KMT2A* protein, encoded by the gene located on chromosome 11q23, is produced via proteolytic cleavage of nascent *KMT2A* by taspase-1 into N-terminal (*KMT2A-N*) and C-terminal (*KMT2A-C*) fragments. *KMT2A-N* contains domains critical for chromatin interactions with menin-binding sites, AT-hooks and CpG islands, while *KMT2A-C* contains the SET domain responsible for H3K4 methylation [4,5,6]. These two subunits interact with other proteins such as WD repeat protein 5 (WDR5), retinoblastoma binding protein 5 (RbBP5), ASH2L, and DPY30, together forming a *KMT2A* complex [7]. *KMT2A* plays an important role as a transcriptional coactivator, especially in hematopoietic stem cell (HSC) self-renewal and differentiation, by interacting with *HOX* gene clusters [8,9].

*KMT2A* rearrangements represent the more common form of genomic aberrations when compared with tandem duplications, copy number alterations, and mutations in *KMT2A* [8,10]. Most *KMT2A* rearrangements are balanced translocation involving 11q23 that results in the production of in-frame gain-of-function oncogenic fusion proteins [8,11]. In an analysis of 3401 cases of acute leukemia, greater than 100 *KMT2A* fusion partner genes were identified. The most common *KMT2A* fusion partners in AML include *AF9 (MLLT3)*, *AF10 (MLLT10)*, *KMT2A-PTDs*, *ELL*, *AF6 (AFDN)*, *ENL (MLLT1)*, and *MLLT11* [11]. Besides AML, *ENL* (Eleven Nineteen Leukemia)-associated protein (EAP) complex169 also accounts for approximately 90% of *KMT2A-r* acute lymphoblastic leukemia (ALL) [12]. The breakpoints for these fusions mostly occur distal to exon 9 in hematologic malignancies (*KMT2A N*-fusion) resulting in the loss of *PHD* and *SET* domains (contrast with sarcoma where *KMT2A C*-fusion mostly occurs) [4,11]. *KMT2A* fusion proteins interact with H3K79 methyltransferase DOT1L or components of the super elongation complex through their C-terminal partners subsequently enhancing transcription elongation and upregulation of gene mechanisms responsible for inhibition of apoptosis and cellular differentiation [13,14]. These epigenetic interactions also drive abnormal activity of key *KMT2A* targets such as *HOX A/B*, *MEIS1* and *PBX3*, which are normally active in early stem cells and gradually phase out during hematopoietic maturation. When their expression remains high, maturation arrest and leukemogenesis follows [13,14,15].

## 3. NPM1 Structure and Mutations

*NPM1* mutations define another major molecular subset, occurring in about one-third of adults with AML [16,17]. The *NPM1* gene encodes for a multifunctional chaperone and shuttling nucleolar protein. *NPM1* protein is composed of a hydrophobic N-terminal core containing two nuclear export signals (NES), a central acidic core containing bipartite nuclear localization signal (NLS), and a basic C-terminal core containing two tryptophan residues (W288 and W290) and nucleolar localization signal (NoLS) [18,19]. The N terminus acts as a chaperone by preventing protein misfolding in the nucleolus. The negatively charged aspartic and glutamic acids in the central region promote histone chaperone activity, while the NLS mediates nuclear transportation of NPM1 protein. The C-terminus enables interactions with nucleic acid and TP53 and drives nucleolar localization of NPM1 [18,20]. NPM1 also associates with the centrosome and prevents excessive duplication during the cell cycle resting phase [21]. Under nucleolar stress, NPM1 is exported out of the nucleolus to the nucleoplasm, inhibits E3 ligase human double minute 2 (HDM2) activity, raises TP53 levels, and triggers cell cycle arrest and apoptosis [22].

*NPM1* mutations commonly arise within a background of replication errors due to aberrant terminal deoxynucleotidyl transferase activity [23]. This results in a frameshift mutation in C-terminus with subsequent loss of W288 and W290 and formation of a new NES. Both events (as well as rare *NPM1* fusion proteins), coupled with increased recognition of NES by XPO1, upregulate the delocalization of mutant *NPM1* from the nucleoplasm to the cytoplasm, forming the basis for leukemogenesis [18,24,25].

## 4. Menin Pathobiology

**Figure 1:**
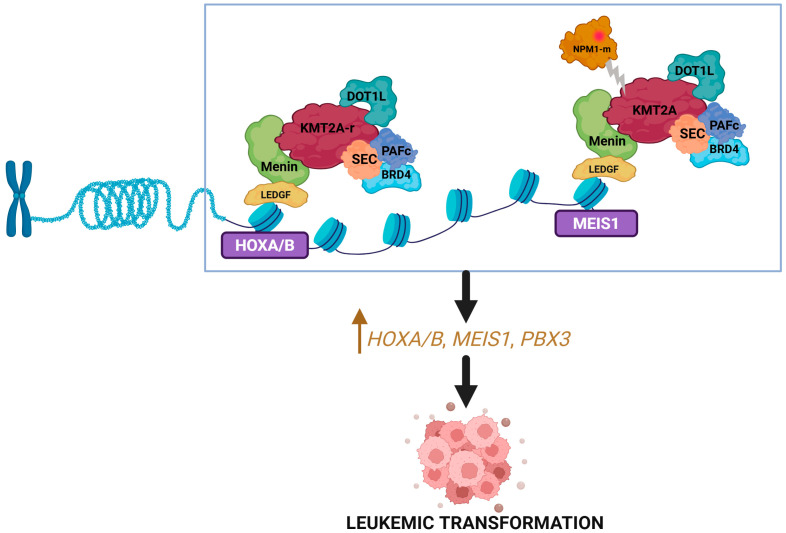
**Menin and *KMT2A* interaction**. *KMT2A* binds with menin to form a complex that upregulates the expression of *HOX* genes and their cofactor *MEIS1* resulting in leukemogenesis. Mutated NPM1 interacts with the menin–KMT2A complex to promote similar downstream effects. Created in BioRender. Joshi, U. (2026) https://BioRender.com/kmznz3i.

Menin is a scaffold or adaptor protein that is encoded by *MEN1* gene and plays an important role in epigenetic regulation via its interaction with multiple partner genes [26]. Menin serves as a bridge between *KMT2A* (wild type or rearranged) and lens-epithelium-derived growth factor (*LEDGF*) and interacts with other transcription factors such as arginine methyltransferase 5 (PRMT5) and the H3K9 methyltransferase SUV39H1 (suppressor of variegation 3–9 homolog 1) [27,28]. Through these interactions, menin has been shown to upregulate prooncogenic transcriptional mechanisms mediated by *KMT2A* and support downstream maintenance of *HOXA*, *MEIS1*, and *PBX3* clusters (Figure 1) [26,27]. Preclinical studies on *KMT2A-r* leukemia models have shown the inhibition of menin to result in the elimination of the leukemia phenotype and restoration of normal hematopoietic maturation [29].

Notably, the gene expression profile of *NPM1-m* AML mimics that of *KMT2A-r* AML, with increased expression of *HOXA/B* and *MEIS1* [30]. This stems from the interaction between mutant NPM1 protein and the menin–*KMT2A* complex. As a result, mutant NPM1 protein nuclear relocation or targeted degradation, as well as inhibition of menin, can all reduce *HOX/MEIS1* expression and inhibit leukemogenesis [30,31].

Beyond *KMT2A-r* and *NPM1-m* disease, there are other subsets of AML that may respond to menin inhibitors. *NUP98*-fusion proteins have been shown to interact with *KMT2A* and associate with a high *HOXA*-expressing AML [32]. When applied to this genomic subgroup, menin inhibition can similarly disrupt the menin–KMT2A interaction, *NUP98*-fusion protein, and chromatin. Other genetic subsets where menin inhibitors have shown some promise in preclinical studies include AML with *UBTF* tandem duplication, *RUNX1/SETD1* co-mutation, and *CEBPA/RUNX1* co-mutation [33].

## 5. Menin Inhibition

### 5.1. Mechanism of Action

**Figure 2:**
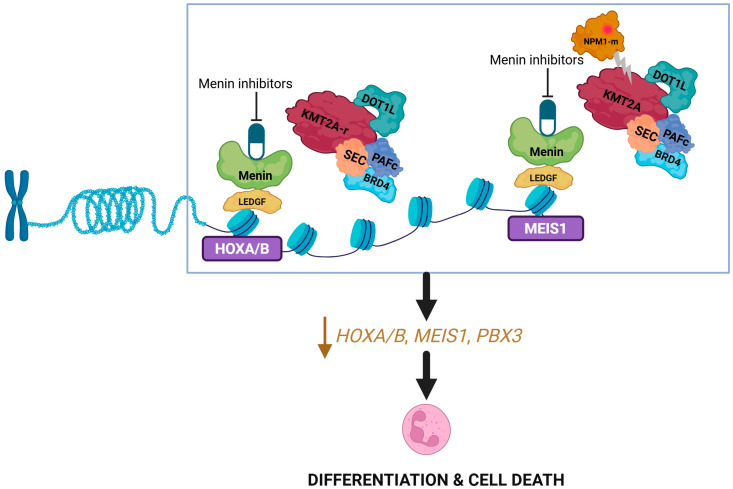
**Mechanism of action of menin inhibitors**. Disruption of the menin–KMT2A complex by menin inhibitors leads to downregulation of the HOX genes. Created in BioRender. Joshi, U. (2026) https://BioRender.com/ogfch57.

Menin inhibitors are small molecule inhibitors of protein-protein interaction that selectively bind to menin binding motif 1 pocket and block interaction between menin and *KMT2A* (and other proteins like *NPM1*) [34]. This results in the downregulation of the *HOXA/MEIS1/PBX3* gene cluster responsible for leukemogenesis and the upregulation of CD11b/CD14 genes responsible for differentiation (Figure 2) [35]. Revumenib is a first-in-class, orally administered small molecule menin inhibitor, and our discussion of the pharmacology of this drug class will be based on revumenib as the reference agent.

### 5.2. Pharmacokinetics

The available menin inhibitors and those currently under study differ in their pharmacokinetic properties. As discussed below, revumenib has been thoroughly studied in patients with relapsed/refractory (R/R) acute leukemia with *KMT2A-r* or *NPM1-m*. Revumenib has a steady-state volume of distribution of 63 L and is approximately 90% protein bound, independent of plasma concentration. Elimination occurs primarily via feces (approximately 52%, with 7% as unchanged drug) and the urine (approximately 25%, with 6% as unchanged drug) [36]. Metabolism is strongly dependent on the concomitant use of cytochrome P450 3A4 (CYP3A4) inhibitors [37]. When administered with a strong CYP3A4 inhibitor at 160 mg twice daily, revumenib reaches a maximum plasma concentration (Cmax) of 3028 ng/mL, a median time to peak (Tmax) of 2 h, a half-life of 6.4 h, and a clearance of 7 L/hour. Without CYP3A4 inhibition, at a dose of 270 mg twice daily, the Cmax is 2344 ng/mL, Tmax is 1 h, the half-life is 3 h, and clearance is 20 L/hour [35,36,37]. These interactions are particularly relevant in patients receiving triazole antifungals such as posaconazole or voriconazole, which are strong CYP3A4 inhibitors and can increase the Cmax and area under curve (AUC) of revumenib by roughly two-fold [36]. In addition, patients weighing under 40 kg exhibit higher drug exposure; therefore, dosing is adjusted according to body surface area in this group [37]. Pharmacokinetic parameters are otherwise consistent across age, race, sex, and mild to moderate hepatic or renal impairment [34,36]. Data are lacking for patients with severe hepatic (total bilirubin > 3× upper limit of normal and any AST) or renal impairment (creatinine clearance < 15 mL/min). There are no data on the central nervous system (CNS) drug penetration or efficacy, so continuation of standard intrathecal therapy is recommended for individuals with known CNS involvement or elevated risk, in keeping with institutional practice.

Ziftomenib is another menin inhibitor with a comparable mechanism of action but distinctive pharmacokinetic features. It has a median Tmax of 3.5 h and an elimination half-life of 61.5 h, indicating rapid absorption and supporting once-daily dosing. Its absolute bioavailability is 12.9%. Ziftomenib undergoes limited metabolism through oxidation, N-demethylation, and N-dealkylation, and is predominantly excreted unchanged in the feces [38].

Enzomenib (DPS-5336) was specifically designed to be different than other currently available/studied menin inhibitors, namely with a short half-life of 2–5 h, low lipophilicity and high clearance; these properties are hypothesized to predict a different therapeutic window for relevant patient populations [39].

### 5.3. Mechanisms of Resistance

**Figure 3:**
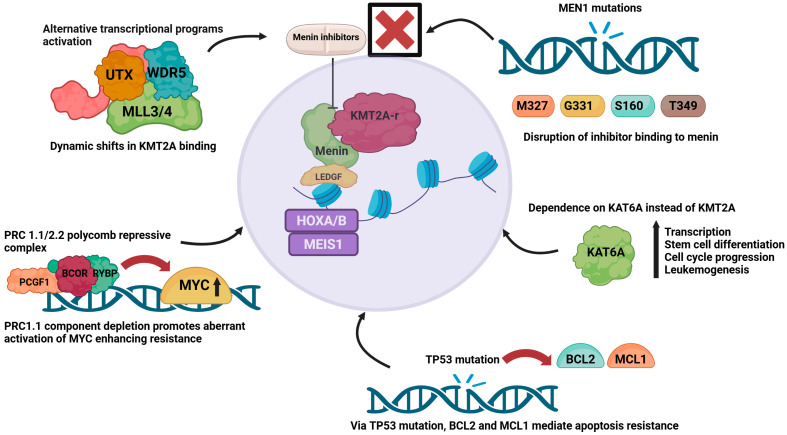
**Mechanism of resistance to menin inhibitors**. Overview of key mechanisms of resistance, including transcriptional reprogramming via MLL3/4–UTX, shift to KAT6A dependency, Polycomb complex dysregulation with MYC activation, TP53-driven apoptosis resistance, and MEN1 mutations that impair inhibitor binding. Created in BioRender. Joshi, U. (2026) https://BioRender.com/o3yaq58.

The development of resistance represents a significant therapeutic challenge for menin inhibitor therapy (Figure 3). Leukemia cells with *KMT2A-r* or *NPM1-m* can acquire somatic changes in *MEN1* that reduce the effectiveness of menin inhibitors. These alterations commonly affect residues M327, G331, T349, and S160, which subsequently weaken the ability of menin to bind small-molecule inhibitors, but without affecting its interaction with *KMT2A*. As a result, the menin–*KMT2A* complex continues to engage with chromatin and drive leukemogenic gene expression, despite menin inhibitor exposure [40]. Additional resistance mechanisms have been described, including *TP53* alterations and their influence on dysregulated BH3 family proteins such as BCL-2 and MCL-1, as well as epigenetic adaptations driven by *KMT2A* fusion oncoproteins, although the understanding of these pathways requires further study [41,42]. The activation of alternative transcriptional programs driven by the MLL3/4–UTX complex leads to dynamic shifts in KMT2A oncoprotein binding, promotes lineage plasticity, and induces a consequent reduction in susceptibility to menin inhibition [43]. In addition, leukemic cells can reduce their reliance on KMT2A by becoming dependent on the histone acetyltransferase KAT6A, which helps sustain high levels of transcription, supports hematopoietic differentiation, drives cell cycle progression, and maintains leukemogenic potential [44]. Resistance is further promoted by disruptions in Polycomb repressive complexes, particularly PRC1.1 and PRC2.2. Loss of PRC1.1 components such as PCGF1, BCOR, and RYBP results in the inappropriate activation of oncogenic pathways, including MYC, a key gene regulated by both the menin–KMT2A axis and PRC1.1 [45,46]. Particular menin inhibitors appear to have activity and efficacy in the presence of specific *MEN1* mutations, as well as mutations in *FLT3* and *IDH1/2*, as discussed below.

## 6. Revumenib

### 6.1. Efficacy of Monotherapy for Relapsed/Refractory Disease

Revumenib (Revuforj^®^, Syndax Pharmaceuticals) is the most extensively studied menin inhibitor and has now been approved by U.S. Food and Drug Administration (FDA) for management of R/R acute leukemia with *KMT2A* rearrangement or *NPM1* mutation [36]. The efficacy of revumenib was demonstrated in the phase I/II AUGMENT-101 study, which evaluated patients with *KMT2A-r* or *NPM1-m* disease [35,47]. The efficacy cohort of *KMT2A-r* acute leukemia (*N* = 57) included heavily pretreated patients with approximately 70% treated with ≥2 prior lines of treatment and 45% with prior hematopoietic cell transplant (HCT), 86% with AML and 12% with ALL, and 80% with R/R disease. The overall response rate (ORR) was 63.2%, with a median time to first response of just under one month. The rate of complete response (CR) or complete response with partial hematologic recovery (CRh) was 22.8%, achieved at a median of 1.9 months. The median duration of CR/CRh was 6.4 months. Of 10 evaluable patients with CR/CRh who had minimal residual disease (MRD) data available, 70% were MRD negative at a median of 1.08 months. Of those who achieved composite complete response (CRc), 68% (15/22) were MRD negative. The median overall survival (OS) was eight months, and 38.9% subsequently received allogeneic HCT [35].

The efficacy cohort of *NPM1-m* AML (*N* = 64) also included heavily pretreated R/R patients, with 36% having received ≥3 prior therapy. in total, 75% were treated with venetoclax, 47% with *FLT3* inhibitors, and 22% had undergone HCT (4% had >1 prior HCT). The ORR was 46.9%, with a median time to first response of 1.84 months. The rate of CR/CRh was 23.4%, with a median time to first CR/CRh of 2.7 months and a median duration of CR/CRh of 4.7 months. Among responders, 16.7% (5/30) subsequently received allogeneic HCT. Median event-free survival (EFS) and OS of the entire cohort were three and four months, respectively. For those who achieved CR/CRh, median OS was 23.3 months. Patients with venetoclax-naïve disease and those with *IDH1/2* co mutations achieved higher rates of CR/CRh [47].

### 6.2. Efficacy of Combination Therapy for Relapsed/Refractory Disease

Revumenib has also shown encouraging activity in combination regimens. In the pediatric AUGMENT-102 study (*N* = 27), which paired revumenib with fludarabine and cytarabine in patients with R/R acute leukemia with *KMT2A-r*, *NPM1-m*, or *NUP98* rearrangement, CRc was achieved in 55.6% at dose level 1 (113 mg twice daily) and 50% at dose level 2 (163 mg twice daily). The median duration of response was 2.6 months at dose level 1, while it had not yet been reached at dose level 2. Among those with CRc, 71.4% achieved MRD negativity [48].

The SAVE trial is evaluating the efficacy of combination treatment with revumenib, venetoclax and decitabine/cedazuridine, including in R/R AML (*N* = 26). The ORR was 88%, CR/CRh was 58%, and MRD negativity by flow cytometry among those with CR/CRh was 93%. After a median follow-up period of 6.6 months, 6-month relapse-free survival (RFS) and OS were 59% and 74%, respectively [49]. Lastly, the combination of revumenib and the FLT3 inhibitor gilteritinib is being studied with initial results demonstrating QTc prolongation as would be expected given the combination, but surmountable with avoidance of concurrent strong CYP3A4 inhibition [50].

### 6.3. Efficacy in the Frontline Setting

The SAVE trial (NCT05360160) is also studying the less-intensive triplet combination of revumenib, decitabine/cedazuridine, and venetoclax as a frontline approach for patients who are ineligible for high-intensity chemotherapy. The cohort (*N* = 17) presented with a high-risk profile: 35% had adverse-risk disease, and 24% were diagnosed with secondary or therapy-related disease. Furthermore, most patients exhibited co-mutations in signaling or myelodysplasia-related genes. The efficacy data are highly encouraging, with an ORR of 94% and a CR rate of 88%. Notably, all patients achieving CR also attained MRD negativity via flow cytometry. After a median follow-up of six months, the median OS and EFS had not yet been reached [51].

Similarly, the Beat AML Master Trial (BAMT) is evaluating the safety and efficacy of revumenib in combination with azacitidine and venetoclax in adults older than 60 years with newly diagnosed *KMT2A-r* or *NPM1-m* AML (*N* = 43). The ORR, CRc, and CR rates were 88.4%, 81.4%, and 67.4%, respectively. All these rates were numerically superior in the *KMT2A-r* group compared to the *NPM1-m* group. Median time to first response was 28 days, and 84% achieved a marrow remission during the first cycle of treatment. All patients who underwent MRD assessment were negative according to flow MRD, whereas 31% of *NPM1-m* AML patients evaluable for *NPM1* MRD via next generation sequencing (NGS) were MRD negative. With a median follow-up period of 6.9 months, median EFS, median OS, and one-year OS were 13.3 months, 15.5 months, and 62.9%, respectively. Median OS for *NPM1-m* and *KMT2A-r* groups were 15.5 months and 18 months, respectively [52].

The addition of revumenib to intensive frontline chemotherapy regimens is currently being explored in the single-arm SNDX-5613-0708 trial (NCT06226571). Initial data from the seven patients enrolled at Dose Level 1 of this Phase I study show that both the ORR and CR rates were 100% at the time of data cutoff. Furthermore, all patients who achieved CR were found to be MRD negative based on local assessment. A significant proportion of the patients (4/7) have successfully proceeded to HCT [53].

These preliminary data with combination strategies in both newly diagnosed and R/R leukemia are very impressive and suggest the need to further pursue large-scale randomized trials of doublet or triplet combinations of revumenib and chemotherapy.

### 6.4. Safety and Toxicity Profile

The safety profile of revumenib is primarily characterized by differentiation syndrome (DS) and QTc prolongation. Revumenib had DS rates of 27.7% (grade ≥3 16%) in the *KMT2A-r* cohort and 19% (grade ≥3 13.1%) in the *NPM1-r* cohort of the AUGMENT-101 trial, 19% (grade ≥3 5%) in BAMT, and 4% (grade ≥3 4%) in the SAVE trial [35,47,49,52]. Median time to initial onset and median duration of the initial event of DS were 5–10 days and 12–15 days, respectively [35,47,52]. QTc prolongation was another notable concern with revumenib, reported in 42.9% (grade ≥3 22.6%) of patients in *NPM1-m* cohort and 25.5% (grade ≥3 16%) in *KMT2A-r* cohort of the AUGMENT-101 trial, 58% (grade ≥3 8%) in the SAVE trial, and 44% (grade ≥3 12%) in BAMT [35,47,49,52]. Treatment-emergent adverse events (TEAEs) were reported in 98% of patients in AUGMENT-101, with 91% experiencing grade ≥3 TEAEs. Most common grade ≥3 TEAEs apart from DS and QTc prolongation included febrile neutropenia, cytopenia, sepsis including pneumonia, hypokalemia, acute kidney injury, and transaminase elevations [35,47,48,49,52].

## 7. Ziftomenib

### 7.1. Monotherapy for Relapsed/Refractory Disease

Ziftomenib (Komzifti^®^, Kura Oncology) is another menin inhibitor that blocks the formation of menin–*KMT2A* complex and suppresses the oncogenic activity associated with *NPM1-m*. Its clinical development has been led by the phase I/II KOMET-001 trial in adults with R/R AML harboring *KMT2A-r* or *NPM1-m*. In the phase I sub-study, patients were heavily pretreated, with a median of three prior lines of therapies; 69% had prior venetoclax exposure and 31% had previously undergone allogeneic HCT. In the *NPM1-m* cohort (200 mg or 600 mg doses), the ORR was 42%, CR/CRh rate was 31%, and median duration of CR/CRh was 6.6 months. Median OS was 3.5 months (2.7 months with 200 mg dose and 5.6 months with 600 mg dose). In the *KMT2A-r* cohort, the ORR was 9%, CR/CRh rate was 6%, and median duration of CR/CRh was 2.1 months. In post hoc analysis, frequency of DS was noted to be higher in *KMT2A-r* than *NPM1-m* group, and often of a worse grade [54]. Accordingly, the phase II portion focused solely on *NPM1-m* AML. The ORR was 33%, with a median time to first response of 1.9 months. The CR/CRh rate was 22%, with a median time to first CR/CRh of 2.8 months and a median duration of CR/CRh of 3.7 months. Among CR/CRh responders with samples available for central NGS, 61% achieved MRD negativity. Median OS was 6.6 months and reached 18.4 months among responders. Prior venetoclax exposure did not significantly alter CR/CRh rates [55]. *FLT3-ITD* co mutations were associated with lower CR/CRh rates, whereas *IDH1/2* co mutations correlated with higher CR/CRh rates, mirroring observations from revumenib studies [47,55].

### 7.2. Combination Therapy for Relapsed/Refractory Disease

The ongoing Phase I KOMET-007 study (NCT05735184) is evaluating ziftomenib in combination with standard, less-intensive therapy (azacitidine and venetoclax) for patients with R/R *NPM1-m* and *KMT2A-r* AML. Interim analysis, utilizing the 600 mg daily dose of ziftomenib, demonstrated encouraging clinical activity across both cohorts. The *NPM1-m* cohort showed particularly robust results, achieving an ORR of 65% and a CRc rate of 49%. In comparison, the *KMT2A-r* cohort had an ORR of 33% and a CRc rate of 22%. The depth of response was significant, with a high proportion of tested CRc responders achieving MRD negativity (50% for *NPM1-m* and 60% for *KMT2A-r*). At data cutoff, median OS was not yet estimable for the *NPM1-m* group but was 21.1 weeks for the *KMT2A-r* cohort [56].

### 7.3. Frontline Combination Therapy

Beyond the R/R setting, KOMET-007 study also includes a phase Ib cohort investigating ziftomenib as a component of frontline, non-intensive therapy. Specifically, patients with newly diagnosed *NPM1-m* AML ineligible for intensive chemotherapy received ziftomenib (600 mg once daily) combined with standard doses of azacitidine and venetoclax. Initial data (*N* = 39 total; *N* = 31 evaluable for response as of 25 June 2025) demonstrate robust activity with an ORR of 94%, CR rate of 58% and a CRc rate of 84%. A significant proportion of tested CRc responders achieved MRD negativity (54%). After a median follow-up of 16.3 weeks, the median OS has not yet been reached [57].

These encouraging results have directly led to the launch of the placebo-controlled, randomized, phase III KOMET-017 trial (NCT07007312), which is currently recruiting patients with newly diagnosed *NPM1-m* or *KMT2A-r* AML to study the clinical benefit of adding ziftomenib to standard frontline treatment with “7 + 3” (amongst intensive-induction-eligible patients) or azacitidine + venetoclax (amongst intensive-induction-inappropriate patients). For the intensive induction group, primary endpoints include EFS and the rate of MRD-negative CR (*NPM1-m* cohort only). In the non-intensive treatment group, primary endpoints focus on OS and CR rate per European Leukemia Net (ELN) 2022 criteria.

Ziftomenib is also current being studied in combination with quizartinib (NCT06769490) and as a monotherapy (NCT06930352) in patients with newly diagnosed AML deemed to be “unfit” to receive other forms of AML-directed therapy [58,59].

### 7.4. Safety and Toxicity Profile

TEAEs were reported in all patients treated with ziftomenib, with grade ≥ 3 events reported in 93%. Common grade ≥3 TEAEs included febrile neutropenia, cytopenia, pneumonia, DS, sepsis, and hypokalemia. DS occurred in 25% of patients in the phase II cohort (grade ≥3 15%). Ziftomenib was associated with lower rates of QTc prolongation (13%, grade ≥3 9%) and myelosuppression compared with revumenib, an attribute that may make it particularly suitable for older adults or individuals with cardiac comorbidities [54,55].

## 8. Bleximenib

Bleximenib (JNJ-75276617, Johnson & Johnson) is another selective small molecule that disrupts the menin–KMT2A complex and has shown potent antileukemia activity against AML and ALL cell lines in vitro. In mouse models, bleximenib exerted synergistic effects with gilteritinib and with azacitidine/venetoclax in *KMT2A-r* AML cells and, more importantly, it has also demonstrated activity in models harboring *MEN1* mutations such as *M327I* and *T349M* that confer resistance to revumenib [60]. In a first-in-human, phase I study enrolling 56 adults with R/R acute leukemia and *KMT2A-r* or *NPM1-m*, grade ≥3 adverse events occurred in 29% of patients, primarily neutropenia (10%), anemia and thrombocytopenia (7%), DS (5%) and transaminase elevations (3%). Among evaluable patients, 39% had a ≥50% reduction in bone marrow blasts. At a dose of 90 mg twice daily (*N* = 8), the ORR was 50%, including CR/CRh and complete response with incomplete hematologic recovery (CRi) in four patients. Among those receiving ≥45 mg twice daily (*N*  =  20), the ORR was 40%, including CR/CRh/CRi in seven patients. The average time to response (PR or better) was 1.81 months, and time to CR/CRh/CRi was 1.77 months [61].

Bleximenib is being studied in combination with venetoclax as a doublet therapy on the phase Ib ALE1002 dose-finding study (NCT05453903). Early results from 13 patients (including 3 *KMT2A-r* and 10 *NPM1-m* AML cases) demonstrated promising activity, with an ORR of 69.2% and a CR/CRh rate of 23.1%. A notable difference was observed based on prior treatment exposure: venetoclax-naïve patients achieved substantially better CRc rates compared to venetoclax-exposed patients (57.1% vs. 16.7%) [62].

Bleximenib is also being studied in the frontline setting with intensive “7 + 3” in a phase 1b trial (NCT05453903). Preliminary data from 24 evaluable patients indicate high rates of response, with an ORR of 95.8%, a CRc rate of 87.5%, and a CR/CRh rate of 75%. At the time of data cutoff, the median duration of response had not yet been reached [63].

These early phase results were promising enough to prompt the launch of the randomized, phase III, double-blind, placebo-controlled cAMeLot-2 trial (NCT06852222), which will evaluate the addition of bleximenib to azacitidine and venetoclax, with OS and CR rate serving as the primary endpoints [64]. The sister trial for intensive therapy-eligible patients is evaluating the combination of bleximenib with “7 + 3” in the phase 3 HOVON 181 AML/AMLSG 37-25 study (NCT07223814), with EFS selected as the primary endpoint [65].

## 9. Enzomenib

Enzomenib (DSP-5336, Sumitomo Pharma) is a menin inhibitor under investigation in a phase I/II monotherapy study in patients with R/R acute leukemia. As of March 2025, 116 heavily pre-treated patients (2 median prior treatment, 36 patients with prior HCT) with *KMT2A-r*, *NPM1-m*, or *HOXA9/MEIS1*-driven leukemia groups had been treated. There was no dose-limiting toxicity (DLT) or grade > 3 QTc prolongation, and DS occurred in 12.9% without associated mortality. Among patients with *KMT2A-r* (treated concomitantly with azoles), the ORR and CR/CRh rates at various doses of 200, 300, and 400 mg twice daily were 50% and 16.7%, 72.7% and 45.5%, and 75% and 25%, respectively. Median OS for all patients with *KMT2A-r* and treated with enzomenib at a dose of ≥200 mg twice daily was 11.4 months. Among 17 patients with *NPM1-m* AML, the ORR was 58.8% and CR/CRh was 47%. Median OS for all patients treated at 200–400 mg twice daily was 8.5 months [39]. Recent cell line experiments have also elucidated possible synergistic enzomenib-inclusive combinations that may inform phase I trial design(s). Specifically, the data demonstrated that enzomenib exhibits significant synergistic anti-leukemic activity when co-administered with a CDK9 inhibitor (e.g., BAY1251152). This combination is particularly compelling as it maintained high efficacy against menin-inhibitor-resistant AML models [66].

## 10. BN104

An emerging product is BN104 (Servier/BioNova Pharmaceuticals). This agent is being studied in a phase I/II study with initial results from the first 20 patients with R/R AML (3 median prior lines of treatment, 20% with prior HCT) and *KMT2A-r*, *NPM1-m* or *NUP98* rearrangement demonstrating that 35% of patients had grade ≥3 adverse events, most commonly febrile neutropenia (15%) and pneumonia (10%). Grade 1 QT prolongation and grade 2 DS were each observed in 10% patients. Among 11 evaluable patients, the ORR was 88.9% and the CR/CRh was 33.3%, supporting further clinical investigation [67].

## 11. BMF-219

BMF-219 (Biomea Fusion) was the first agent developed associated with covalent and irreversible menin inhibition. It was evaluated in the phase I COVALENT-101 study, which includes patients with R/R acute leukemia, diffuse large B cell lymphoma, multiple myeloma, and chronic lymphocytic leukemia. As of 26 July 2023, patients with R/R acute leukemia (24 AML and 2 ALL) had been treated. Patients were heavily pre-treated, with a median of four prior therapies and 11 having undergone HCT. The drug was well tolerated with no DLT or treatment discontinuations. DS was the only grade ≥3 toxicity, occurring in 13%. Of five evaluable patients for efficacy, one each achieved CR and CRi [68]. The study was eventually terminated and BMF-219 is no longer being studied in hematology/oncology indications.

Comprehensive clinical data for the individual agents and their combinations are presented in Table 1 and Table 2.

## 12. Future Directions

The advent of menin inhibitors represents one of the most pivotal periods of AML drug development and their application has favorably impacted many patients with the disease. Akin to most other AML products, efficacy in the R/R setting has eventually beget studies in earlier lines, typically in combination with reference standards of care. The striking rates and depths of remission are associating with survival estimates that have confidently fostered the launch of several randomized, placebo-controlled, phase III studies in combination with standard intensive induction (i.e., “7 + 3”) or less-intensive therapy (i.e., azacitidine + venetoclax). Pending the results of these phase III studies, the impressive efficacy observed in single-arm studies will increase the use of menin inhibitors, not only in the R/R setting as monotherapy, but also in off-label combinations in both the R/R and frontline settings. The latter being more likely for patients with *KMT2A-r* AML for whom lesser rates of higher-quality remission and higher rates of relapse may warrant accepting more risk of being exposed to off-label combinations that may eventually be proven to be no better than standard therapy upon phase III read outs. Further study of resistance mechanisms and how to overcome them or sequence menin inhibitors is necessary. We expect more menin inhibitor combinations with other targeted agents (e.g., FLT3 and IDH inhibitors) to be studied given the non-low rates of co-mutation amongst patients with *NPM1-m* disease and lack of clarity about which lesion/interaction is best targeted in the R/R setting for such patients. As menin inhibitor use increases, particularly without myelosuppressive backbones and in the community, awareness, vigilance and early intervention for DS is necessary to navigate patients through the critical risk period so that patients destined for response may realize it.

## 13. Conclusions

Menin inhibition has emerged as a novel strategy for leukemias driven by *KMT2A* rearrangements and *NPM1* mutations, as well as other rarer lesions associated with *HOX/MEIS* overexpression. Revumenib, ziftomenib, bleximenib, enzomenib and newer agents (e.g., BN104) have shown meaningful activity across this disease spectrum, with combination approaches being further studied to improve response depth. At the same time, resistance, particularly through *MEN1* mutations, remains a significant therapeutic challenge and has already informed the development of next-generation compounds. Taken together, current data position menin inhibitors as an important addition to targeted therapy in AML, with ongoing trials expected to define their role in routine frontline and salvage treatment.

## Figures and Tables

**Table 1:** Response rate and survival outcomes with Menin inhibitors monotherapy.

Study	Genes	Menin Inhibitors	*N*	Response Rate	EFS	OS
AUGMENT-101 [35]	*KMT2A-r*, R/R	Revumenib	57	ORR 63.2%, CR/CRh 22.8%, CRc 43.9%		Median OS: 8 months
AUGMENT-101 [43]	*NPM1-m*, R/R	Revumenib	64	ORR 46.9%, CR/CRh 23.4%, CRc 29.7%	Median EFS: 3 months	Median OS: 4 months
KOMET-001 [50]	*KMT2A-r*, R/R	Ziftomenib	32 ^#^	ORR 9%, CR/CRh 6%, CRc 9%		Median OS: 5.4 months
KOMET-001 [50,51]	*NPM1-m*, R/R	Ziftomenib	112 *	ORR 35%, CR/CRh 24%, CRc 29%		Median OS: 18.4 months (ORR responders), 3.5 months (non-responders)
75276617 ALE-1001 [57]	*KMT2A-r*, *NPM1-m*, R/R	Bleximenib	20 ^$^, 8 ^%^	ORR ^%^ 50%, CR/CRh ^%^ 25%, ORR 40% ^$^, CR/CRh ^$^ 20%		
NCT04988555 [39]	*KMT2Ar*, *NPM1m*, R/R	Enzomenib	29 ^&^, 17 ^@^	ORR ^&^ 69%, CR/CRh ^&^ 31%, ORR ^@^ 58.8%, CR/CRh ^@^ 47%		Median OS: 11.4 months (*KMT2A-r*), median OS: 8.5 months (*NPM1-m*)
NCT06052813 [63]	*KMT2Ar*, *NPM1m*, *NUP98-r* R/R	BN104	11	ORR 88.9%, CR/CRh 33.3%		

^#^ Includes patients on both 200 or 600 mg ziftomenib. * Includes pooled patient data from phase Ib and II of KOMET-001 study. ^$^ Includes ≥45 mg BID cohort with disease evaluation data available. ^%^ Includes 90 mg BID cohort with disease evaluation data available. ^&^ Includes patients with *KMT2A*-r and evaluable data for efficacy (doses of 200, 300, and 400 mg BID). ^@^ Includes patients with *NPM1*-m and evaluable data for efficacy (doses of 200 or 300 mg BID). *KMT2A-r*: histone-lysine N-methyltransferase 2A rearrangements, *NPM1-m*: Nucleophosmin 1 mutated, R/R: relapsed/refractory, ORR: overall response rate, CR: Complete response rate, CRh: Complete response with partial hematologic recovery, CRc: Composite complete response, EFS: event free survival, OS: Overall survival.

**Table 2:** Response rate and survival outcomes with menin inhibitors in combination.

Study	Genes	Menin Inhibitors Combination	*N*	Response Rate	EFS	OS
AUGMENT-102 [44]	*KMT2A-r*, *NPM1-m*, *NUP98-r*, R/R	Revumenib, fludarabine, cytarabine	27	CRc 55.6% (DL1), 50% (DL2)		
SAVE [45]	*KMT2A-r*, *NUP98-r*, *NPM1-m*, R/R	Revumenib, venetoclax, decitabine/cedazuridine	26	ORR 88%, CR/CRh 58%, CRc 43.9%	6-month RFS: 59%	6-month OS: 74%
NCT06222580 [46]	*KMT2A-r*, *NUP98-r*, *NPM1-m + FLT3-m*, R/R	Revumenib, gilteritinib	7 ^#^	MLFS 66.6% (DL0), CR 33.3% (DL1)		
SAVE [47]	*KMT2A-r*, *NUP98-r*, *NPM1-m*, 1L	Revumenib, venetoclax, decitabine/cedazuridine	17	ORR 94%, CR 88%	Median EFS: NR *	Median OS: NR *
Beat AML Master Trial [48]	*KMT2A-r*, *NPM1-m*, 1L	Revumenib, venetoclax, azacitidine	43	ORR 88.4%, CR 67.4%, CRc 81.4%	Median EFS: 13.3 months	Median OS: 15.5 months, 1-year OS: 62.9%
SNDX-5613-0708 [49]	*KMT2A-r*, *NUP98-r*, *NPM1-m*, 1L	Revumenib, cytarabine and daunorubicin or idarubicin	7	ORR/CR/CRc 100%		
KOMET-007 [52]	*KMT2A-r*, *NPM1-m* R/R	Ziftomenib, azacitidine, venetoclax	70	ORR 65% ^$^ and 33% ^&^, CRc 49% ^$^ and 22% ^&^		Median OS: NR ^$^ and 21.1 weeks ^&^
KOMET-007 [53]	*NPM1-m*, 1L	Ziftomenib, azacitidine, venetoclax	31	ORR 94%, CR 58%, CRc 84%		Median OS: NR
ALE1002 [58]	*KMT2A-r*, *NPM1-m*, R/R	Bleximenib, venetoclax	13	ORR 69.2%, CR/CRh 23.1%, CRc 38.5%		
ALE1002 [59]	*KMT2Ar*, *NPM1m*, 1L	Bleximenib, cytarabine and daunorubicin or idarubicin	24	ORR 95.8%, CR/CRh 75%, CRc 87.5%		
NCT06052813 [63]	*KMT2Ar*, *NPM1m*, *NUP98-r* R/R	BN104	11	ORR 88.9%, CR/CRh 33.3%		

^#^ Total *N* = 7, but only 6 patients evaluable at the time of data presentation. * Median EFS and OS not reached at a median follow-up of 6 months. ^$^ Response rates and OS for *NPM1-m* group. ^&^ Response rates and OS for *KMT2A-r* group. *KMT2A-r*: histone-lysine N-methyltransferase 2A rearrangements, *NPM1-m*: Nucleophosmin 1 mutated, R/R: relapsed/refractory, ORR: overall response rate, CR: Complete response rate, CRh: Complete response with partial hematologic recovery, CRc: Composite complete response, MLFS: Morphologic leukemia free state, DL: dose level, NR: not reached, EFS: event free survival, OS: Overall survival.

## Data Availability

No new data were created or analyzed in this study. Data sharing is not applicable to this article.

## Abbreviations

The following abbreviations are used in this manuscript:

AML	Acute myeloid leukemia
KMT2A	Histone-lysine N-methyltransferase 2A
*KMT2A*-r	KMT2A rearrangements
NPM1	Nucleophosmin 1
*NPM1*-m	NPM1 mutated
HOX	Homeobox
MEIS1	Myeloid ecotropic virus insertion site 1
MLL	Mixed-lineage leukemia
H3K4	Histone three lysine 4
SET	Su(var)3–9, Enhancer of zester and Trithorax
WDR5	WD repeat protein 5
RbBP5	Retinoblastoma binding protein 5
HSC	Hematopoietic stem cell
EAP	Eleven Nineteen Leukemia-associated protein
ALL	Acute lymphoblastic leukemia
NES	Nuclear export signals
NLS	Nuclear localization signal
NoLS	Nucleolar localization signal
HDM2	Human double minute 2
LEDGF	Lens epithelium-derived growth factor
SUV39H1	Suppressor of variegation 3–9 homolog 1
R/R	Relapsed/refractory
CYP3A4	Cytochrome P450 3A4
Cmax	Maximum plasma concentration
Tmax	Time to peak
AUC	Area under curve
CNS	Central nervous system
FDA	Food and Drug Administration
HCT	Hematopoietic cell transplant
ORR	Overall response rate
CR	Complete response
CRh	Complete response with partial hematologic recovery
MRD	Minimal residual disease
OS	Overall survival
CRc	Composite complete response
EFS	Event free survival
RFS	Relapse free survival
BAMT	Beat AML Master Trial
NGS	Next generation sequencing
TEAE	Treatment emergent adverse events
DS	Differentiation syndrome
ELN	European Leukemia Net
DLT	Dose limiting toxicity
MLFS	Morphologic leukemia free state
DL	Dose level
NR	Not reached
